# Prediction of Postoperative Venous Thromboembolism in Patients With Traumatic Brain Injury: Model Development and Validation Study

**DOI:** 10.2196/78655

**Published:** 2025-11-17

**Authors:** Jiang Zheng, Qiling Jiang, Yusheng Zhan, Yanming Tang, Xiaohui Du, Guangrong Xiang, Yufang Ouyang, Hong Fu

**Affiliations:** 1 Department of Anesthesiology, Chongqing Emergency Medical Center, Chongqing University Central Hospital, School of Medicine, Chongqing University, Chongqing, China Chongqing China; 2 The First People's Hospital of Chongqing High-Tech Zone Chongqing China

**Keywords:** venous thromboembolism, traumatic brain injury, machine learning, postoperative, predictive model

## Abstract

**Background:**

Venous thromboembolism (VTE) remains a critical cause of mortality among patients who are hospitalized. Patients with traumatic brain injury (TBI) are particularly susceptible to VTE due to coagulation abnormalities and immobilization. Despite this elevated risk, no validated predictive model currently exists for postoperative VTE in populations with TBI.

**Objective:**

This study aims to develop machine learning (ML)–based predictive models for VTE in patients with TBI undergoing surgical procedures, with a focus on clinical translatability.

**Methods:**

Data were collected from patients with TBI who underwent surgical treatment at Chongqing University Central Hospital (from October 2016 to December 2024). The dataset was randomly partitioned into a training set and an internal test set in a 7:3 ratio. The recursive feature elimination algorithm was applied for feature selection, followed by the synthetic minority oversampling technique to address class imbalance. Six ML models, including logistic regression (LR), random forest, gradient boosting decision tree, extreme gradient boosting, support vector machine, and categorical boosting, were trained and validated. Model performance was evaluated using receiver operating characteristic analysis, calibration curves (assessing probability-observation alignment), and decision curve analysis to quantify clinical net benefit. For the LR model, clinical utility was enhanced through nomogram construction, with Shapley additive explanation values providing interpretability.

**Results:**

A total of 1806 participants were enrolled in this study, and 257 (14.2%) experienced VTE events. All ML models demonstrated strong predictive performance, with area under the receiver operating characteristic curve values ranging from 0.79 to 0.83. The LR model exhibited the highest discriminatory power (area under the receiver operating characteristic curve 0.83; accuracy 0.80; specificity 0.83). Calibration curves confirmed that the LR model provided well-calibrated probability estimates. Shapley additive explanations analysis identified key contributors to VTE risk and transformed model outputs into individualized risk predictions. A user-friendly postoperative VTE risk prediction nomogram was developed for patients with TBI.

**Conclusions:**

This study successfully developed and validated multiple ML models for postoperative VTE prediction in patients with TBI. The LR-based nomogram, supported by calibration and decision curve validation, offers a clinically actionable tool to guide thromboprophylaxis strategies. Future external validation across diverse populations is warranted to confirm generalizability.

## Introduction

Venous thromboembolism (VTE), encompassing deep vein thrombosis (DVT) and pulmonary embolism (PE), is a critical clinical syndrome associated with high morbidity and mortality among hospitalized patients [1]. Globally, an estimated 10 million individuals experience VTE annually. In the United States, PE ranks among the leading causes of cardiovascular death, accounting for up to 300,000 fatalities per year [2]. Patients with VTE experience severe complications, including adverse effects of long-term anticoagulation (eg, hemorrhage risk), prolonged hospitalization, elevated 30-day readmission rates, and delayed adjuvant therapies (eg, chemotherapy or radiotherapy) [3]. VTE significantly contributes to extended hospital stays and increased mortality [4,5]. Notably, patients with traumatic brain injury (TBI) who develop VTE exhibit prolonged intensive care unit stays and extended mechanical ventilation duration [6].

TBI affects approximately 50 million individuals globally each year, with epidemiological models suggesting that nearly half of the global population may sustain at least 1 TBI during their lifetime [7]. TBI is an independent risk factor for VTE, elevating the risk through multifactorial pathophysiological mechanisms [6]. Studies indicate that patients with multitrauma and TBI face significantly higher VTE risk than those without TBI, attributable to disease complexity and delayed early interventions [8]. Coagulopathy is prevalent in this population; 67% of the patients with severe TBI exhibit coagulation abnormalities upon emergency presentation, which often progress during hospitalization [9,10]. In addition, cohorts of patients who underwent surgical procedures demonstrate elevated VTE incidence due to venous stasis from general anesthesia and intraoperative immobilization, postoperative mobility restrictions, and tissue injury–induced inflammation and coagulation pathway activation [11].

Severe TBI is strongly associated with coagulopathy, substantially elevating the risk of VTE, particularly in cohorts of patients who underwent surgical procedures. Identifying patients at high risk for postoperative VTE is thus critically imperative. Despite this established clinical need, current literature lacks dedicated studies developing VTE predictive models tailored to patients with surgical TBI. Machine learning (ML) has demonstrated significant efficacy in perioperative risk prediction across diverse surgical contexts [12]. Therefore, the objective of our study is to develop an ML-based risk assessment model for VTE in patients with TBI undergoing surgical procedures.

## Methods

### Recruitment

This retrospective study consecutively enrolled patients hospitalized with TBI who underwent surgical intervention at Chongqing University Central Hospital between October 2016 and December 2024.

### Ethical Considerations

This study was approved by the Institutional Review Board of Chongqing University Central Hospital (2024-66). The requirement of obtaining written informed consent was waived owing to the retrospective study design. All visual representations in this manuscript, including feature importance diagrams, contain only aggregated or anonymized data. No personally identifiable information of research participants is displayed in any figures or supplementary materials.

### Data Collection

#### Overview

This study collected comprehensive clinical data from patients with TBI who underwent surgery, encompassing 6 core domains.

#### Demographics

Demographic data included age, sex, weight, height, American Society of Anesthesiologists (ASA) physical status classification, and history of smoking and alcohol use.

#### Preoperative Comorbidities

Preoperative comorbidities included hypertension, coronary heart disease, diabetes mellitus, atrial fibrillation, chronic renal insufficiency, myocardial infarction, pulmonary infection, and malignant tumors.

#### Injury Characteristics

The following variables were recorded: Glasgow Coma Scale (GCS) score, Rotterdam computed tomography score, injury mechanism (falls, traffic accidents, or assaults), Injury Severity Score, Abbreviated Injury Scale (AIS), head injury types (eg, concussion, cerebral contusion, diffuse axonal injury, subdural or epidural hematoma, subarachnoid hemorrhage, or skull fracture), and injury-to-admission interval.

#### Biochemical Parameters

Biochemical parameters included complete blood count, liver function tests, comprehensive metabolic panel, and coagulation profiles.

#### Preoperative Interventions

Information on medications and supportive measures included the following: anticoagulants and antiplatelets (eg, rivaroxaban, clopidogrel, and aspirin), hemostatic agents (eg, tranexamic acid, vitamin K, and etamsylate), and supportive therapies (eg, pneumatic compression therapy, mechanical ventilation, blood transfusion, endotracheal intubation or tracheostomy, and mannitol administration).

#### Surgical and Anesthesia Details

Data included surgical site (intracranial or extracranial), emergency status, multiple surgeries (≥2 procedures), anesthesia method (general or regional), surgery duration, intraoperative fluid balance, blood loss volume, vasopressor use, invasive monitoring (central venous), and intraoperative hypotension (systolic blood pressure <90 mm Hg).

Missing data were imputed using the K-nearest neighbors algorithm for continuous variables and the mode for categorical variables.

### Outcome

The diagnosis of VTE, encompassing DVT and PE, relies on confirmatory imaging studies integrated with clinical presentation. The first-line imaging modality for DVT is Doppler ultrasonography, which identifies noncompressible veins with hypoechoic thrombi and abnormal blood flow patterns. Symptoms of PE typically present acutely and include respiratory and cardiovascular manifestations. The diagnosis of PE is commonly established using computed tomography pulmonary angiography. Clinical signs suggestive of DVT in the lower limbs include edema, discomfort, tenderness, the presence of a palpable cord, and erythema or cyanosis.

**Inclusion and Exclusion Criteria**

The inclusion and exclusion criteria are presented in Textbox 1.

**Inclusion and exclusion criteria.**

**Inclusion Criteria**

- Patients with traumatic brain injury (including concussion, cerebral contusion, diffuse axonal injury, subarachnoid hemorrhage, and epidural or subdural hemorrhage of traumatic origin)
- Patients undergoing surgical management during the current hospitalization
- Patients aged 18 years or older at the time of enrollment

**Exclusion Criteria**

- Patients aged younger than 18 years
- No surgical intervention performed during the study period, surgery performed before admission to the study institution, or surgery conducted during previous hospitalizations
- Missing more than 20% of essential study variables
- Length of hospital stay shorter than 72 hours after admission
- Venous thromboembolism diagnosed before surgery

### Model Development and Explanation

The comprehensive dataset was stratified randomly and partitioned into a training set (1264/1806, 70%) and an internal validation set (542/1806, 30%) while preserving class distribution. Feature selection was performed using the recursive feature elimination algorithm followed by application of the synthetic minority oversampling technique to mitigate class imbalance [13,14]. A total of 6 ML algorithms were evaluated for predictive performance: logistic regression (LR), support vector machine (SVM), random forest (RF), gradient boosting decision tree (GBDT), extreme gradient boosting (XGBoost), and categorical boosting (CatBoost).

Model performance was comprehensively evaluated through a suite of discriminative, calibrated, and clinically interpretable metrics. For discriminative capacity, we assessed receiver operating characteristic analysis with area under the curve, alongside precision-recall metrics (including *F*_1_-score—the harmonic mean of precision and recall), sensitivity, specificity, positive predictive values (PPVs), and negative predictive values (NPVs). Calibration was quantified using the Brier score and visualized via calibration curves that compared predicted probabilities against observed outcomes across risk deciles to ensure reliable probability estimates. In terms of clinical interpretability, Shapley additive explanations (SHAP) were applied to quantify feature contributions to individual predictions, elucidating both global feature importance and local decision logic, while a nomogram was developed based on LR coefficients to translate model outputs into clinically actionable risk stratification categories. Advanced validation further strengthened the robustness of findings. Decision curve analysis was performed to evaluate net clinical benefit across threshold probabilities (0%-100%), comparing model-guided interventions against “treat-all” or baseline strategies.

### Statistical Analysis

Continuous variables were presented as medians (IQRs), and categorical variables were presented as numbers and percentages. All analyses were performed using PyCharm (version 2023.3.4; JetBrains), SPSS (version 26.0; IBM Corp), and R (version 4.2.1; R Foundation for Statistical Computing).

## Results

### Overview

**Figure 1:**
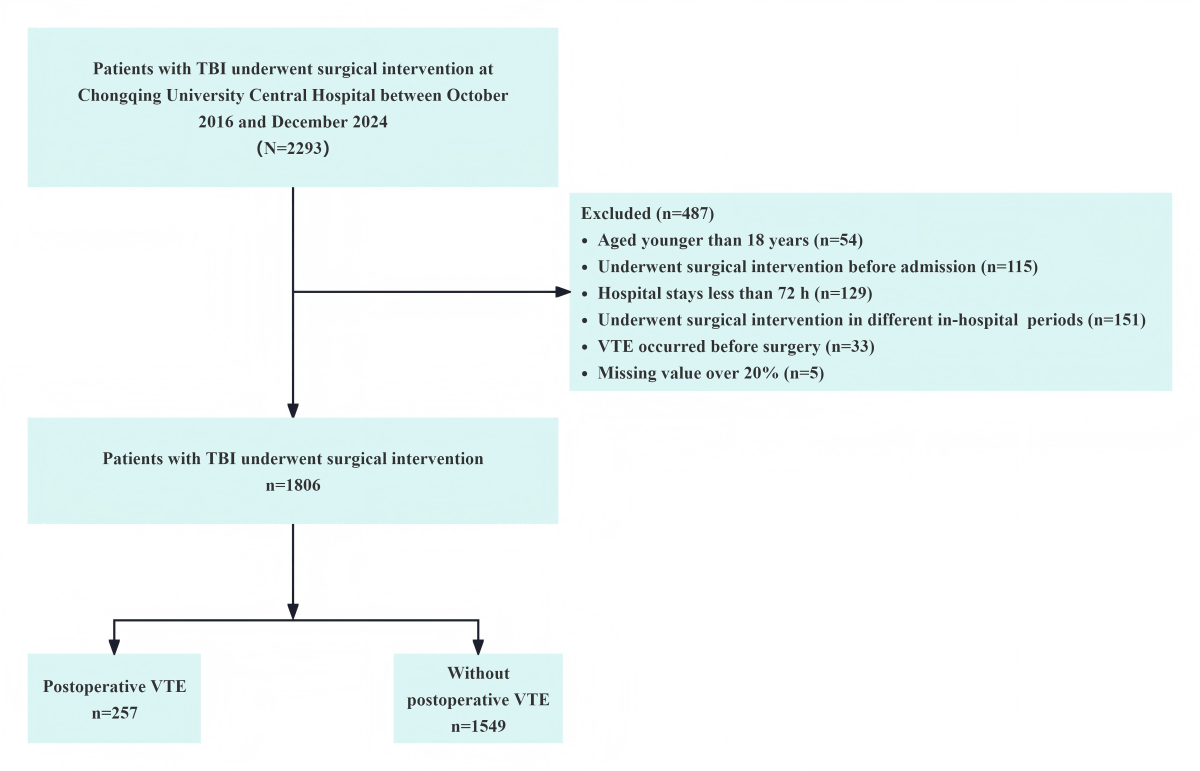
The flowchart of the study population. TBI: traumatic brain injury; VTE: venous thromboembolism.

From October 2016 to December 2024, a total of 1806 patients with TBI who underwent surgical intervention were enrolled in this study (Figure 1). Among these patients, 257 (14.2%) experienced VTE, including 254 (14.1%) cases of DVT and 3 (0.2%) cases of PE (Figure S1 in Multimedia Appendix 1). Table 1 presents the demographic and clinical characteristics of the study population. The median age of the patients was 51 (IQR 37-62) years. A total of 1324 (73.4%) patients were male. The most common cause of injury was traffic accidents, affecting 1033 (57.2%) patients. Cerebral contusion was diagnosed in 1017 (56.3%) patients, subdural or epidural hematoma in 967 (53.5%) patients, and subarachnoid hemorrhage in 910 (50.4%) patients (Figures S2 and S3 in Multimedia Appendix 1). Regarding the severity of head injury based on the GCS scores, 1022 (56.6%) patients had mild head injury (GCS score 13-15), 226 (12.5%) had moderate head injury (GCS score 9-12), and 558 (30.9%) had severe head injury (GCS score 3-8; Table 1 and Figure S4 in Multimedia Appendix 1). For the modeling dataset, 1806 cases were included, with 1264 (70%) assigned to the training set and 541 (30%) to the internal validation set (Table 1).

**Figure 2:**
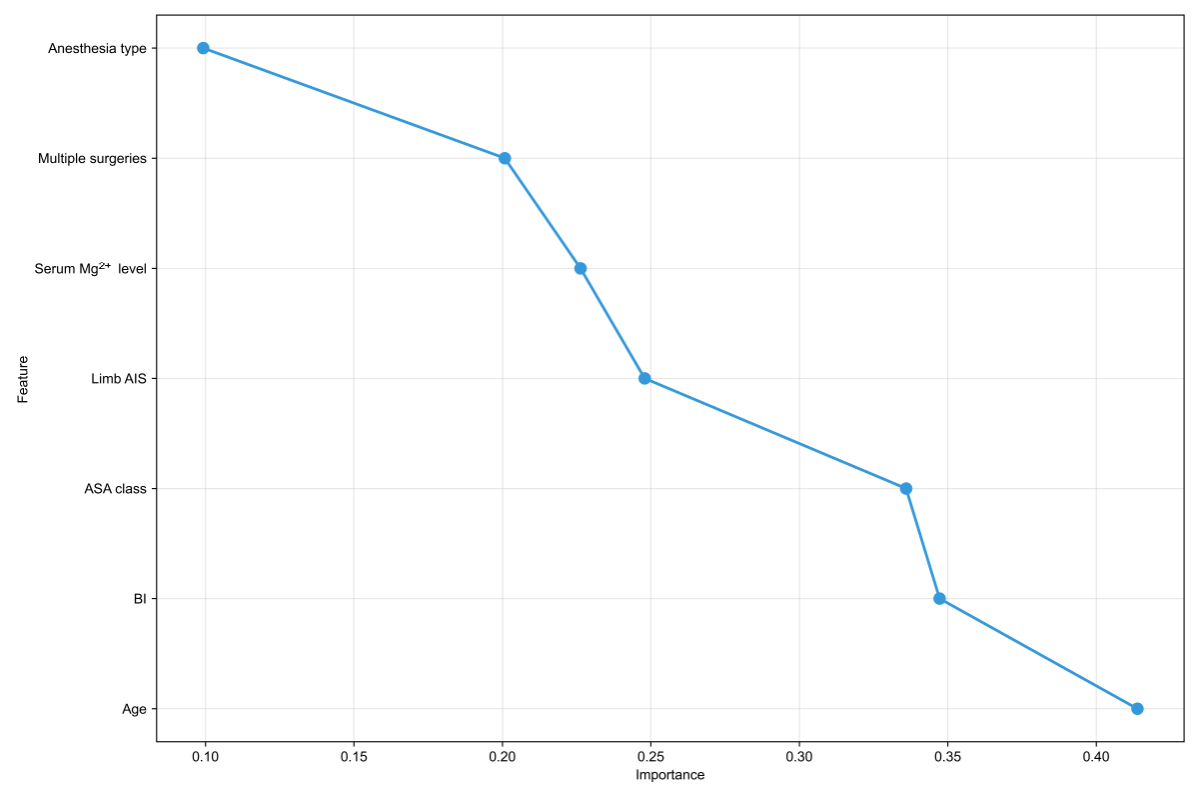
Selected features by recursive feature elimination. AIS: Abbreviated Injury Scale; ASA: American Society of Anesthesiologists; BI: Barthel index; Mg: magnesium.

The top 7 variables selected for inclusion in the models based on the recursive feature elimination were as follows: age, Barthel index (BI), ASA class, multiple surgeries, anesthesia types, serum magnesium (Mg^2+^) levels, and limb AIS score (Figure 2).

**Table 1:** Baseline characteristics of the patients in the training set and test set.

Variables		Total (N=1806)	Training set (n=1264)	Test set (n=542)
Age (y), median (IQR)		51 (37-62)	52 (37-61)	51 (36-62)
Weight (kg), median (IQR)		62 (55-70)	63 (55-70)	62 (55-70)
Barthel index, median (IQR)		0 (10-30)	10 (0-30)	10 (0-30)
Male, n (%)		1324 (73.3)	935 (74)	389 (71.8)
ASA^a^ physical status classification≥3, n (%)		1133 (62.7)	800 (63.2)	333 (61.4)
Delayed admission, n (%)		278 (15.4)	197 (15.6)	82 (15.1)
Smoking, n (%)		539 (29.8)	384 (30.4)	155 (28.6)
Drinking, n (%)		288 (15.9)	201 (15.9)	87 (16.1)
Hypertension, n (%)		236 (13.1)	166 (13.1)	70 (12.9)
Coronary heart disease, n (%)		35 (1.9)	22 (1.7)	13 (2.4)
Diabetes, n (%)		112 (6.2)	83 (6.6)	29 (5.4)
Pulmonary infection, n (%)		66 (3.7)	45 (3.6)	21 (3.9)
Chronic kidney disease, n (%)		10 (0.6)	7 (0.6)	3 (0.6)
Cancer, n (%)		14 (0.8)	11 (0.9)	3 (0.6)
Atrial fibrillation, n (%)		16 (0.9)	12 (0.9)	4 (0.7)
Estrogen or progestin, n (%)		16 (0.9)	8 (0.6)	8 (1.5)
Shock, n (%)		189 (10.5)	127 (10)	62 (11.4)
**Preoperative treatment, n (%)**				
	Admission to the ICU^b^	861 (47.7)	593 (46.9)	268 (49.4)
	Anticoagulation and antiplatelet therapy	474 (26.2)	429 (33.9)	145 (26.8)
	Hemostatic drugs	173 (9.6)	109 (8.6)	64 (11.8)
	Mannitol	485 (26.9)	353 (27.9)	133 (24.5)
	Trachea intubation	154 (8.5)	106 (8.4)	48 (8.9)
	Tracheotomy	66 (3.7)	41 (3.2)	25 (4.6)
	Limb pressure therapy	80 (4.4)	64 (5.1)	17 (3.1)
	Mechanical ventilation	136 (7.5)	92 (7.3)	44 (8.1)
	Blood transfusion	116 (6.4)	75 (5.9)	42 (7.7)
**Mechanism of injury, n (%)**				
	Fall	293 (16.2)	211 (16.7)	82 (15.1)
	Traffic accident	1033 (57.2)	706 (55.9)	326 (60.1)
	Fall from height	354 (19.6)	256 (20.3)	98 (18.1)
	Assault	85 (4.7)	67 (5.3)	18 (3.3)
	Attack	41 (2.3)	23 (1.8)	18 (3.3)
**Type of head injury**				
	Concussion, n (%)	403 (22.3)	287 (22.7)	116 (21.4)
	Contusion, n (%)	1017 (56.3)	712 (56.3)	305 (56.3)
	Subdural or extradural hematoma, n (%)	967 (53.5)	676 (53.5)	291 (53.7)
	Diffuse axonal injury, n (%)	87 (4.8)	64 (5.1)	23 (4.2)
	Subarachnoid hemorrhage, n (%)	910 (50.4)	643 (50.9)	267 (49.3)
	Skull fracture, n (%)	684 (37.9)	469 (37.1)	215 (39.7)
	ISS^c^, median (IQR)	16 (11-22)	16 (10-22)	16 (11-22)
	Head AIS^d^ score, median (IQR)	3 (3-3)	3 (2-3)	3 (2-3)
	Limb AIS score, median (IQR)	0 (0-3)	0 (0-2)	0 (0-2)
	GCS^e^ score, median (IQR)	13 (7-15)	13 (7-15)	13 (8-15)
	Mild, n (%)	1022 (56.6)	711 (56.3)	311 (57.4)
	Moderate, n (%)	226 (12.5)	158 (12.5)	68 (12.5)
	Severe, n (%)	558 (30.9)	395 (31.3)	163 (30.1)
	Rotterdam CT^f^ score, median (IQR)	2 (1-2)	2 (1-2)	2 (1-2)
**Intraoperative events, n (%)**				
	Vasoconstrictor	984 (54.5)	693 (54.8)	292 (53.9)
	Transfusion	336 (18.6)	239 (18.9)	98 (18.1)
	Invasive monitoring	1156 (64)	825 (65.3)	332 (61.3)
	Emergency	949 (52.5)	686 (54.3)	263 (48.5)
	General anesthesia	1589 (88)	1124 (88.9)	466 (86)
	Hypotension	743 (41.1)	508 (40.2)	235 (43.4)
**Surgical site, n (%)**				
	Head	831 (46)	598 (47.3)	245 (45.2)
	Thorax	360 (19.9)	239 (18.9)	121 (22.3)
	Limbs	452 (25)	313 (24.8)	139 (25.6)
	Abdomen	88 (4.9)	68 (5.4)	21 (3.9)
	Body surface	74 (4.1)	58 (4.6)	15 (2.8)
Multiple surgeries, n (%)		489 (27.1)	347 (27.5)	143 (26.4)
Intracranial surgery, n (%)		674 (37.3)	480 (38)	194 (35.8)
Extracranial surgery, n (%)		1268 (70.2)	880 (69.6)	389 (71.8)
Operation duration (min), median (IQR)		158 (109-215)	157 (106-210)	160 (110-220)
Bleeding (mL), median (IQR)		200 (50-500)	200 (50-500)	200 (93-400)
Intraoperative fluid volume (mL), median (IQR)		1800 (1200-2600)	1800 (1200-2600)	1700 (1200-2500)

^a^ASA: American Society of Anesthesiologists.

^b^ICU: intensive care unit.

^c^ISS: Injury Severity Score.

^d^AIS: Abbreviated Injury Scale.

^e^GCS: Glasgow Coma Scale.

^f^CT: computed tomography.

### Model Performance

**Figure 3:**
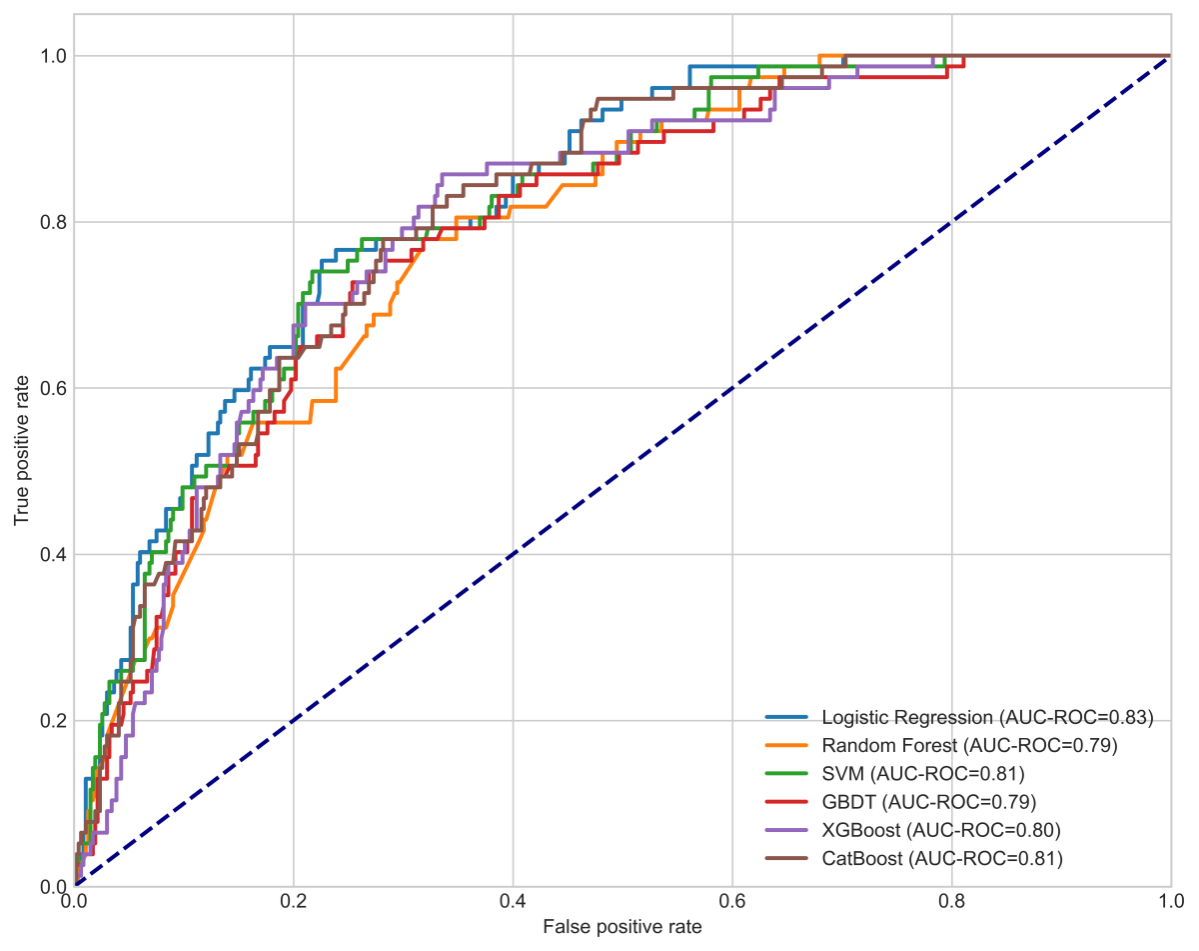
Area under the receiver operating characteristic curves (AUC-ROC) of 6 machine learning models in the internal validation dataset. CatBoost: categorical boosting; GBDT: gradient boosting decision tree; SVM: support vector machine; XGBoost: extreme gradient boosting.

Figure 3 displays the area under the receiver operating characteristic curve (AUC-ROC) for the 6 models in the internal validation set. Table 2 and Figure S5 in Multimedia Appendix 1 present the additional evaluation metrics, including accuracy, sensitivity, specificity, PPV, and NPV for each model.

**Table 2:** Model performance in predicting venous thromboembolism in the validation set.

Model	AUC-ROC^a^	Accuracy	Sensitivity	Specificity	PPV^b^	NPV^c^	*F*_1_-score
Logistic regression	0.83	0.80	0.62	0.83	0.37	0.93	0.47
Random forest	0.79	0.72	0.69	0.72	0.29	0.92	0.41
SVM^d^	0.81	0.78	0.74	0.78	0.36	0.95	0.49
GBDT^e^	0.80	0.77	0.65	0.79	0.34	0.93	0.44
XGBoost^f^	0.80	0.80	0.58	0.84	0.38	0.92	0.45
CatBoost^g^	0.81	0.79	0.57	0.83	0.36	0.92	0.44

^a^AUC-ROC: area under the receiver operating characteristic curve.

^b^PPV: positive predictive value.

^c^NPV: negative predictive value.

^d^SVM: support vector machine.

^e^GBDT: gradient boosting decision tree.

^f^XGBoost: extreme gradient boosting.

^g^CatBoost: categorical boosting.

Among the evaluated models, LR demonstrated superior performance in the internal validation set, achieving the highest AUC-ROC of 0.83, with an accuracy of 0.80 and specificity of 0.83, consistently generating reliable predictions and accurately identifying true negative cases. Although other models showed merit, they fell short in key performance domains. RF had an AUC-ROC of 0.78 (lower than LR), accuracy of 0.72, and specificity of 0.82, but its sensitivity of 0.69 (relatively low) led to more false negatives. SVM had an AUC-ROC of 0.81 (close to LR) but underperformed in sensitivity (0.74) and PPV (0.36). GBDT and XGBoost both achieved an AUC-ROC of 0.80 (respectable but trailing LR), with GBDT’s sensitivity (0.65) slightly better than LR and XGBoost, despite the highest accuracy (0.80) among non-LR models, suffering from suboptimal PPV (0.38) and lower sensitivity (0.58). CatBoost, with an AUC-ROC of 0.81, had a low sensitivity (0.57) despite a high NPV (0.92; still lower than LR’s NPV of 0.93). These results underscore LR’s balanced performance across discriminative, calibration, and clinical utility metrics, making it the most robust choice for VTE prediction in this cohort of patients with TBI undergoing surgical procedures.

### Model Explanation Results and Nomogram

**Figure 4:**
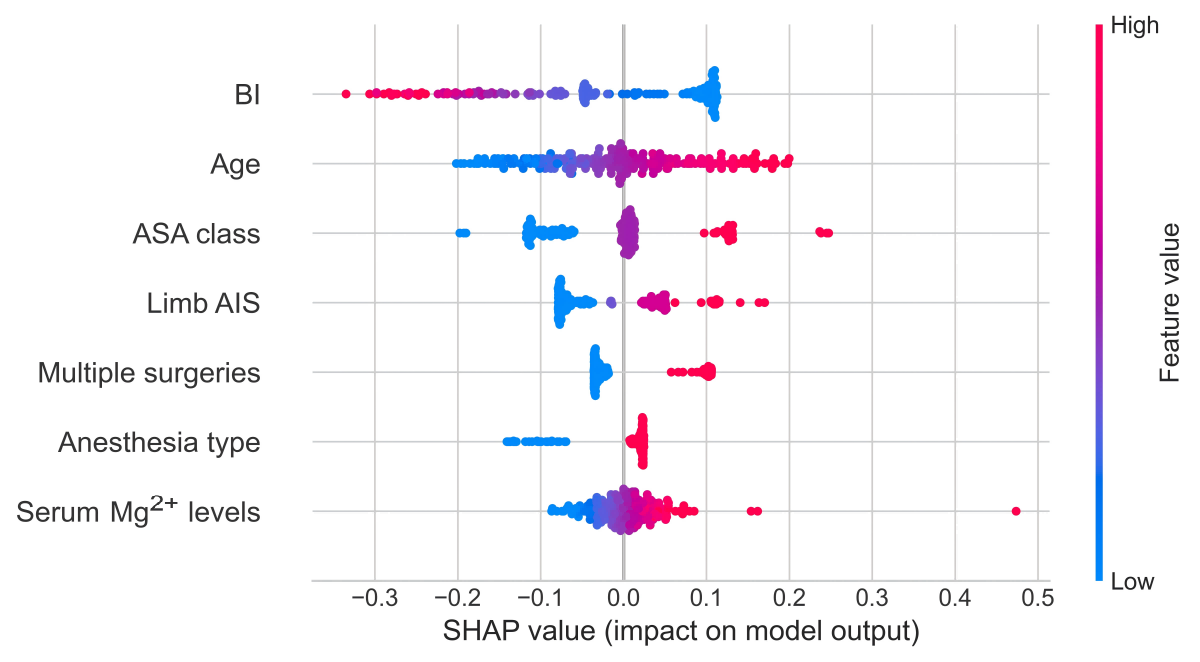
Global feature importance on the logistic regression model. AIS: Abbreviated Injury Scale; ASA: American Society of Anesthesiologists; BI: Barthel Index; Mg: magnesium; SHAP: Shapley additive explanations.

The SHAP algorithm enabled interpretable insights at both global and instance-specific levels. Figure 4 visualizes the relative importance and directional impact of 7 key features on the LR model’s predictions, as derived from SHAP’s interpretation of the model’s output. These features were identified as critical predictors: BI, age, limb AIS, ASA Class, multiple surgeries, serum magnesium (Mg^2+^) levels, and anesthesia type.

**Figure 5:**
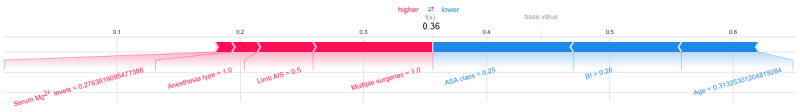
Local explanation for a non–venous thromboembolism sample. AIS: Abbreviated Injury Scale; ASA: American Society of Anesthesiologists; BI: Barthel index; Mg: magnesium.

**Figure 6:**
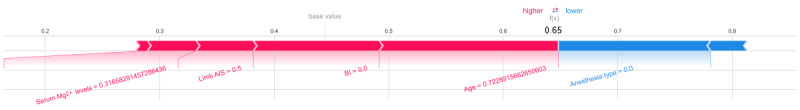
Local explanation for a venous thromboembolism sample. AIS: Abbreviated Injury Scale; ASA: American Society of Anesthesiologists; BI: Barthel index; Mg: magnesium.

Subsequently, we linked SHAP values to their directional impact on VTE risk (ie, the probability of a feature increasing or decreasing the likelihood of VTE) and visualized these relationships using data from 1 patient with VTE and 1 patient without VTE in the internal validation dataset (Figure 5). Figure 6 specifically illustrates a case of a patient with VTE, where red segments denote features with positive SHAP values (indicating they contributed to an increased predicted VTE risk) and blue segments represent features with negative SHAP values (indicating they reduced the predicted VTE risk). This visualization clarified how individual features collectively modulate the model’s risk stratification for VTE.

**Figure 7:**
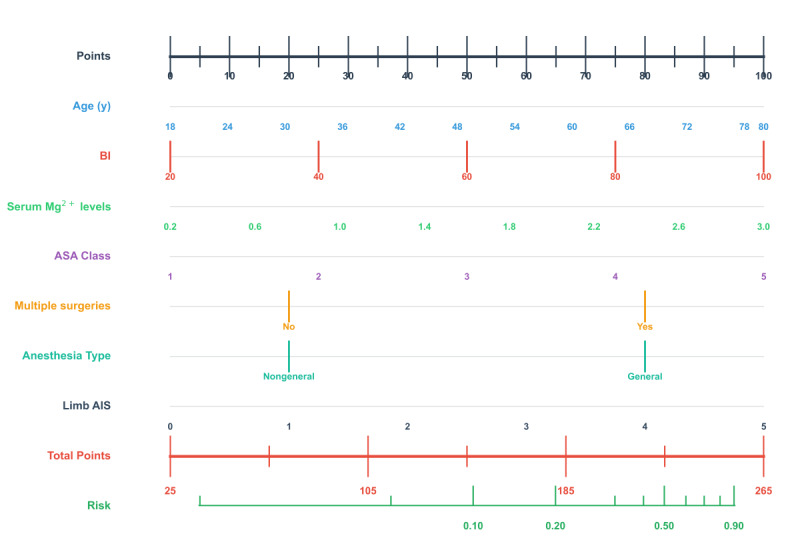
Postoperative venous thromboembolism risk prediction nomogram for patients with traumatic brain injury. AIS: Abbreviated Injury Scale; ASA: American Society of Anesthesiologists; BI: Barthel index; Mg: magnesium.

Figure 7 presents a user-friendly nomogram for rapid risk quantification, where feature scores are weighted by regression coefficients, allowing clinicians to sum points and visualize risk thresholds. This aligned with SHAP-derived feature importance, bridging statistical rigor with clinical decision-making. The calibration curve illustrated the calibration performance of the LR model in the internal validation set, with the x-axis representing predicted probabilities (0-1) and the y-axis depicting observed risks (0-1). Key curves included the bias-corrected curve (derived from 1000 bootstrap resamples), which aligned closely with the ideal reference line in the moderate-risk range (0.2-0.7), indicating strong predictive accuracy and the apparent curve, reflecting raw data fit with minor deviations at extreme probabilities (<0.2 and >0.8; Figure S6 in Multimedia Appendix 1). Further evaluation of clinical utility using the decision curve analysis demonstrated that the nomogram provided superior net benefits on the validation set (Figure S7 in Multimedia Appendix 1).

Collectively, the LR model’s balanced performance (AUC-ROC=0.83; NPV=0.93), robust calibration, and actionable interpretability—coupled with the nomogram’s clinical utility—establish it as a reliable tool for VTE prediction in TBI surgical cohorts.

## Discussion

### Principal Findings

The prevention of VTE remains a cornerstone in the management of patients with TBI, particularly those undergoing surgical intervention, due to their heightened thrombotic risk driven by prolonged immobilization, hypercoagulability, and endothelial injury. Although pharmacological thromboprophylaxis (eg, low-molecular-weight heparin) and mechanical methods (eg, sequential compression devices) are widely implemented, balancing efficacy with safety remains challenging due to the risk of intracranial hemorrhage associated with these agents [15]. Current guidelines lack consensus on optimal dosing protocols, timing of initiation, and patient selection criteria, further complicating clinical decision-making [16].

In this study, we documented a postoperative VTE incidence of 14.2% among patients with TBI, markedly exceeding the 3.9% reported in a 2019 national registry study [17]. This disparity underscores the urgent need for improved risk assessment tools to identify high-risk patients and guide targeted thromboprophylactic interventions.

Previous studies have used various risk-scoring systems to assess PE risk in patients who were hospitalized, including the Caprini and Padua scores [18-20]. The Caprini score, while simple and widely adopted, has significant limitations, such as its overreliance on subjective historical data (eg, previous VTE and obesity), omission of objective biomarkers (eg, D-dimer dynamics), and failure to account for anatomical factors (eg, craniotomy type) or anticoagulation therapy risks. For instance, in Asian cohorts, where patients with TBI frequently undergo prolonged surgeries (>45 min) and present with comorbid conditions (eg, hypertension), the Caprini score often overestimates VTE risk by classifying excessive numbers of patients as high risk. This has led to unnecessary anticoagulation therapy, increasing bleeding risks and health care costs [21,22].

To address these challenges and more accurately identify patients with TBI at high risk of postoperative VTE, we used 6 ML algorithms (including LR, RF, SVM, XGBoost, GBDT, and CatBoost) to develop predictive models and created a postoperative VTE risk prediction nomogram specifically for patients with TBI. These models aim to enhance risk assessment precision, enabling more targeted and effective thromboprophylactic interventions. ML has demonstrated efficacy in VTE prediction across various patient populations. Wang et al [23] and Liu et al [24] validated the RF model for VTE risk assessment in Chinese inpatients and patients who have experienced stroke, respectively.

### Comparison With Prior Work

The morphology and influence of most features on the predictions are consistent with clinical practice and previous evidence. LR-based global feature importance analysis identified several critical VTE risk factors. BI scores, a validated measure of activities of daily living, emerged as the strongest predictor, with poorer functional status (lower BI scores) substantially increasing VTE risk—likely due to immobility and venous stasis—with moderate-certainty evidence supporting this association [25,26]. Advanced age was another key determinant of VTE risk, as older patients exhibited heightened susceptibility to VTE, attributed to age-related coagulation changes, increased comorbidity burden, and potential immobilization—all well-documented VTE risk factors consistent with previous epidemiological studies. Severe limb injuries (eg, pelvic fractures) were significantly associated with elevated VTE risk, aligning with findings by Hereford et al [27]. Higher ASA scores, reflecting greater comorbidity burden and poorer physiological reserve, strongly correlated with increased VTE risk, consistent with the American College of Chest Physicians’ guidelines, which emphasize ASA status in VTE risk assessment for patients undergoing major surgeries [28].

Our study further demonstrated that lower serum magnesium (Mg^2+^) levels are associated with higher VTE risk, potentially mediated by endothelial dysfunction (eg, slowed endothelial cell proliferation, stimulated monocyte adhesion, and impaired synthesis of vasoregulatory molecules) and myocardial instability (eg, altered intracellular calcium handling, prolonged QT intervals, and increased arrhythmia risk) [29-31]. However, a prospective cohort study conducted by Kunutsor and Laukkanen [32] among middle-aged Caucasian men (n=2361) reported no significant association between serum magnesium (Mg^2+^) levels and VTE risk (adjusted hazard ratio 1.38, 95% CI 0.48-3.96), with discrepancies potentially attributed to population heterogeneity (eg, age, sex, and comorbidities). Therefore, future studies are warranted to validate the association between serum magnesium levels and VTE risk in more diverse populations encompassing varying age groups, sexes, and comorbid conditions. Although other factors (eg, multiple surgeries and anesthesia type) also influenced VTE risk to a lesser extent, these findings collectively provide critical insights for developing targeted preventive strategies and enhancing patient-specific risk assessment in clinical practice. This study investigated potential VTE predictors in patients with TBI after undergoing surgery and compared the performance of multiple machine learning algorithms. Nonetheless, the model's reliance solely on preoperative and intraoperative variables, excluding postoperative data such as laboratory tests and therapeutic interventions, potentially constrained its predictive power. Abbasi et al [33] successfully predicted postoperative bleeding events, VTE, and stroke risk in cardiac surgery by integrating preoperative, intraoperative, and postoperative variables, achieving high performance (AUC-ROC=0.92-0.97). Their study demonstrated that including postoperative variables significantly enhanced model performance, with prediction accuracy critically dependent on these data. Thus, future studies should integrate postoperative laboratory tests and therapeutic interventions as key variables to enhance the predictive capability of VTE risk models in populations with TBI.

### Limitations

Several limitations of this study warrant acknowledgment. First, the dataset was derived from a single medical center for an 8-year period, which may have introduced data heterogeneity and unavoidable selection bias. Second, although this study included 1806 cases with a notably high VTE incidence, the number of VTE-positive events remained relatively small. Therefore, larger sample sizes are critical for future studies to enable more precise analyses and the development of robust predictive models. Third, postoperative laboratory tests and treatment regimens were not integrated into the analysis, which may have affected the study outcomes. Finally, given that all data originated from a single center, external validation of the developed model across multiple centers over time is necessary to confirm its generalizability.

### Conclusions

This study successfully developed and validated ML models for VTE risk prediction in patients with TBI undergoing surgery, with a specific focus on clinical translatability. Leveraging the LR model’s robust performance, we constructed a practical nomogram that enables bedside VTE risk assessment using routinely collected preoperative and intraoperative data. This user-friendly tool empowers clinicians to rapidly quantify individualized VTE risk and guide targeted thromboprophylactic decisions at the point of care. Future external validation across diverse clinical settings and patient populations is warranted to confirm the model’s generalizability and ensure its broader applicability in real-world practice.

## Appendix

Multimedia Appendix 1 Overview of study population characteristics, head injury profiles, and model performance for predicting venous thromboembolism.

## Abbreviations

AIS: Abbreviated Injury Scale
ASA: American Society of Anesthesiologists
AUC-ROC: area under the receiver operating characteristic curve
BI: Barthel index
CatBoost: categorical boosting
DVT: deep vein thrombosis
GBDT: gradient boosting decision tree
GCS: Glasgow Coma Scale
LR: logistic regression
ML: machine learning
NPV: negative predictive value
PE: pulmonary embolism
PPV: positive predictive value
RF: random forest
SHAP: Shapley additive explanations
SVM: support vector machine
TBI: traumatic brain injury
VTE: venous thromboembolism
XGBoost: extreme gradient boosting

## Data Availability

The datasets generated or analyzed during this study are not publicly available due to patient privacy concerns and the ethical restrictions imposed by the Institutional Review Board, but are available from the corresponding author on reasonable request.

## References

[1] Buchanan IA, Lin M, Donoho DA, Patel A, Ding L, Amar AP, Giannotta SL, Mack WJ, Attenello F (2019). Predictors of venous thromboembolism after nonemergent craniotomy: a nationwide readmission database analysis. World Neurosurg.

[2] Wendelboe AM, Raskob GE (2016). Global burden of thrombosis: epidemiologic aspects. Circ Res.

[3] Shah MN, Stoev IT, Sanford DE, Gao F, Santiago P, Jaques DP, Dacey RG (2013). Are readmission rates on a neurosurgical service indicators of quality of care?. J Neurosurg.

[4] Geerts WH, Bergqvist D, Pineo GF, Heit JA, Samama CM, Lassen MR, Colwell CW (2008). Prevention of venous thromboembolism: American College of Chest Physicians Evidence-Based Clinical Practice Guidelines (8th Edition). Chest.

[5] Fernandez MM, Hogue S, Preblick R, Kwong WJ (2015). Review of the cost of venous thromboembolism. Clinicoecon Outcomes Res.

[6] Denson K, Morgan D, Cunningham R, Nigliazzo A, Brackett D, Lane M, Smith B, Albrecht R (2007). Incidence of venous thromboembolism in patients with traumatic brain injury. Am J Surg.

[7] Maas AI, Menon DK, Adelson PD, Andelic N, Bell MJ, Belli A, Bragge P, Brazinova A, Büki A, Chesnut RM, Citerio G, Coburn M, Cooper DJ, Crowder AT, Czeiter E, Czosnyka M, Diaz-Arrastia R, Dreier JP, Duhaime A, Ercole A, van Essen TA, Feigin VL, Gao G, Giacino J, Gonzalez-Lara LE, Gruen RL, Gupta D, Hartings JA, Hill S, Jiang J, Ketharanathan N, Kompanje EJO, Lanyon L, Laureys S, Lecky F, Levin H, Lingsma HF, Maegele M, Majdan M, Manley G, Marsteller J, Mascia L, McFadyen C, Mondello S, Newcombe V, Palotie A, Parizel PM, Peul W, Piercy J, Polinder S, Puybasset L, Rasmussen TE, Rossaint R, Smielewski P, Söderberg J, Stanworth SJ, Stein MB, von Steinbüchel N, Stewart W, Steyerberg EW, Stocchetti N, Synnot A, Te Ao B, Tenovuo O, Theadom A, Tibboel D, Videtta W, Wang KK, Williams WH, Wilson L, Yaffe K, InTBIR ParticipantsInvestigators (2017). Traumatic brain injury: integrated approaches to improve prevention, clinical care, and research. Lancet Neurol.

[8] Chen D, Luo J, Zhang C, Tang L, Deng H, Chang T, Xu H, He M, Wan D, Zhang F, Wu M, Qian M, Zhou W, Yin G, Wang W, Dong L, Tang Z (2023). Venous thrombus embolism in polytrauma: special attention to patients with traumatic brain injury. J Clin Med.

[9] Hoyt DB (2004). A clinical review of bleeding dilemmas in trauma. Semin Hematol.

[10] Harhangi BS, Kompanje EJ, Leebeek FW, Maas AI (2008). Coagulation disorders after traumatic brain injury. Acta Neurochir (Wien).

[11] Agnelli G (2004). Prevention of venous thromboembolism in surgical patients. Circulation.

[12] Zhu Y, Liu X, Li Y, Yi B (2024). The applications and prospects of big data in perioperative anesthetic management. Anesthesiol Perioper Sci.

[13] Guyon I, Elisseeff A (2003). An introduction to variable and feature selection. J Mach Learn Res.

[14] Zhu T, Lin Y, Liu Y (2017). Synthetic minority oversampling technique for multiclass imbalance problems. Pattern Recognit.

[15] Skrifvars MB, Bailey M, Presneill J, French C, Nichol A, Little L, Duranteau J, Huet O, Haddad S, Arabi Y, McArthur C, Cooper DJ, Bellomo R, EPO-TBI investigatorsthe ANZICS Clinical Trials Group (2017). Venous thromboembolic events in critically ill traumatic brain injury patients. Intensive Care Med.

[16] Rappold JF, Sheppard FR, Carmichael Ii SP, Cuschieri J, Ley E, Rangel E, Seshadri AJ, Michetti CP (2021). Venous thromboembolism prophylaxis in the trauma intensive care unit: an American Association for the Surgery of Trauma Critical Care Committee clinical consensus document. Trauma Surg Acute Care Open.

[17] Hoffman H, Jalal MS, Chin LS (2019). The risk factors, outcomes, and costs associated with venous thromboembolism after traumatic brain injury: a nationwide analysis. Brain Inj.

[18] Hou L, Hu L, Gao W, Sheng W, Hao Z, Chen Y, Li J (2021). Construction of a risk prediction model for hospital-acquired pulmonary embolism in hospitalized patients. Clin Appl Thromb Hemost.

[19] Wang L, Wei S, Zhou B, Wu S (2021). A nomogram model to predict the venous thromboembolism risk after surgery in patients with gynecological tumors. Thromb Res.

[20] Zhou H, Hu Y, Li X, Wang L, Wang M, Xiao J, Yi Q (2018). Assessment of the risk of venous thromboembolism in medical inpatients using the padua prediction score and caprini risk assessment model. J Atheroscler Thromb.

[21] Bahl V, Hu HM, Henke PK, Wakefield TW, Campbell DA, Caprini JA (2010). A validation study of a retrospective venous thromboembolism risk scoring method. Ann Surg.

[22] Kim MH, Jun KW, Hwang JK, Kim SD, Kim JY, Park SC, Won Y, Yun S, Moon I, Kim J (2019). Venous thromboembolism following abdominal cancer surgery in the Korean population: incidence and validation of a risk assessment model. Ann Surg Oncol.

[23] Wang X, Yang YQ, Liu SH, Hong XY, Sun XF, Shi JH (2020). Comparing different venous thromboembolism risk assessment machine learning models in Chinese patients. J Eval Clin Pract.

[24] Liu L, Li L, Zhou J, Ye Q, Meng D, Xu G (2024). Machine learning-based prediction model of lower extremity deep vein thrombosis after stroke. J Thromb Thrombolysis.

[25] Mahoney FI, BAarthel DW (1965). Functional evaluation: the Barthel index. Md State Med J.

[26] Kelly J, Rudd A, Lewis RR, Coshall C, Moody A, Hunt BJ (2004). Venous thromboembolism after acute ischemic stroke: a prospective study using magnetic resonance direct thrombus imaging. Stroke.

[27] Hereford T, Thrush C, Kimbrough MK (2019). Using injury severity score and abbreviated injury score to determine venous thromboembolism risk. Cureus.

[28] Kearon C, Akl EA, Comerota AJ, Prandoni P, Bounameaux H, Goldhaber SZ, Nelson ME, Wells PS, Gould MK, Dentali F, Crowther M, Kahn SR (2012). Antithrombotic therapy for VTE disease: antithrombotic therapy and prevention of thrombosis, 9th ed: American College of Chest Physicians evidence-based clinical practice guidelines. Chest.

[29] Swaminathan R (2003). Magnesium metabolism and its disorders. Clin Biochem Rev.

[30] Kostov K, Halacheva L (2018). Role of magnesium deficiency in promoting atherosclerosis, endothelial dysfunction, and arterial stiffening as risk factors for hypertension. Int J Mol Sci.

[31] Chakraborti S, Chakraborti T, Mandal M, Mandal A, Das S, Ghosh S (2002). Protective role of magnesium in cardiovascular diseases: a review. Mol Cell Biochem.

[32] Kunutsor SK, Laukkanen JA (2021). Circulating serum magnesium and the risk of venous thromboembolism in men: a long-term prospective cohort study. Pulse (Basel).

[33] Abbasi A, Li C, Dekle M, Bermudez CA, Brodie D, Sellke FW, Sodha NR, Ventetuolo CE, Eickhoff C (2025). Interpretable machine learning-based predictive modeling of patient outcomes following cardiac surgery. J Thorac Cardiovasc Surg.

