# Supplementary material for: Prediction of Postoperative Venous Thromboembolism in Patients With Traumatic Brain Injury: Model Development and Validation Study
Source: JMIR Med Inform. 2025 Nov 17;13:e78655. doi: 10.2196/78655 (PMC12670048; doi:10.2196/78655)

Supplement

Figure S1 The gender distribution of study population

Figure S2 The distribution of injury mechanism

Figure S3 The distribution of head injury types

Figure S4 The distribution of head injury severity

Figure S5 Model performance in predicting venous thromboembolism in the validation set

Figure S6 Calibration plot of the logistic regression model

Figure S7 Decision curve analysis for the nomogram

Figure S1 The gender distribution of study population

Figure S2 The distribution of injury mechanism

Figure S3 The distribution of head injury types

Figure S4 The distribution of head injury severity

Figure S5 Model performance in predicting venous thromboembolism in the validation set

AUC, area under the receiver operating characteristic curve.

PPV, positive predictive value.

NPV, negative predictive value.

SVM, Support Vector Machine.

GBDT, Gradient boosting decision Tree.

XGBoost, extreme gradient boosting.

Categorical Boosting.

Figure S6 Calibration plot for validation cohort


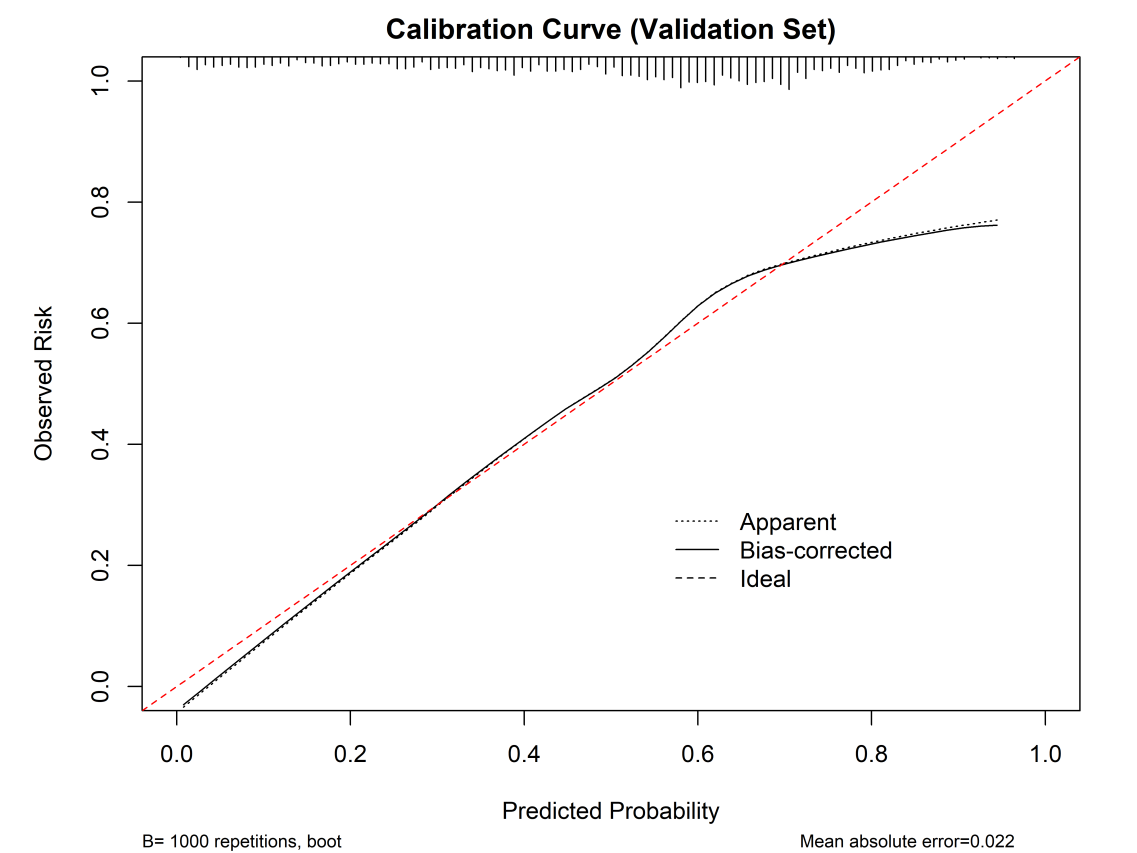


Figure S7 Decision curve analysis for the nomogram


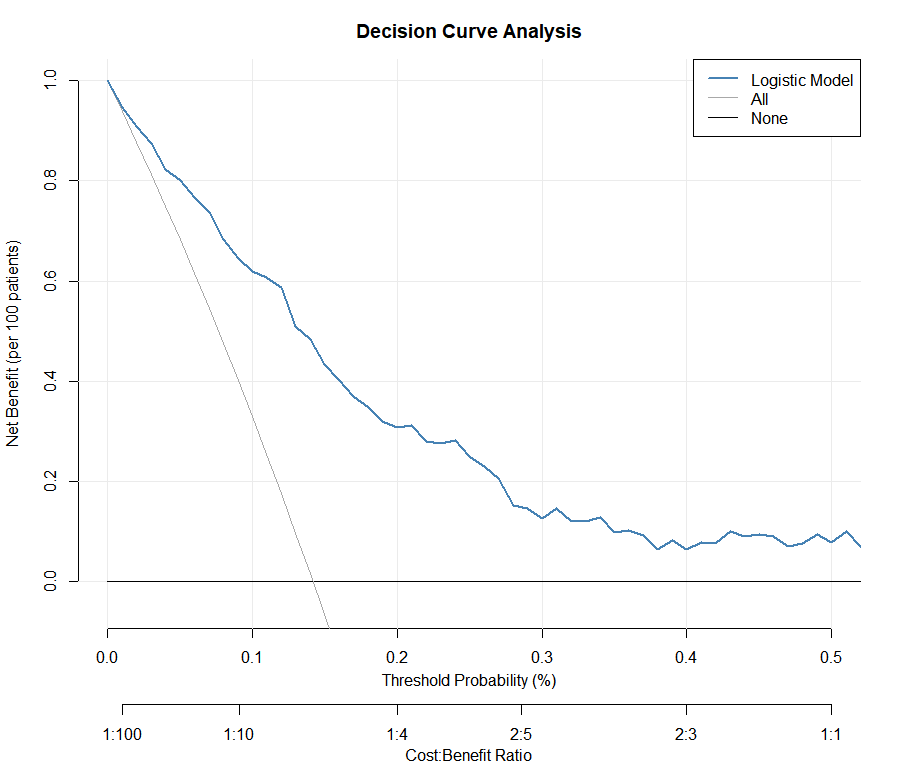

Supplement: Multimedia Appendix 1 [file medinform_v13i1e78655_app1.docx]
